# A mobile phone application for the assessment and management of youth mental health problems in primary care: a randomised controlled trial

**DOI:** 10.1186/1471-2296-12-131

**Published:** 2011-11-29

**Authors:** Sophie C Reid, Sylvia D Kauer, Stephen JC Hearps, Alexander HD Crooke, Angela S Khor, Lena A Sanci, George C Patton

**Affiliations:** 1 Murdoch Childrens Research Institute, University of Melbourne; 2 Royal Children's Hospital, University of Melbourne; 3 Department of General Practice, University of Melbourne; 4 School of Behavioural Science, University of Melbourne, Australia

## Abstract

**Background:**

Over 75% of mental health problems begin in adolescence and primary care has been identified as the target setting for mental health intervention by the World Health Organisation. The *mobiletype *program is a mental health assessment and management mobile phone application which monitors mood, stress, coping strategies, activities, eating, sleeping, exercise patterns, and alcohol and cannabis use at least daily, and transmits this information to general practitioners (GPs) via a secure website in summary format for medical review.

**Methods:**

We conducted a randomised controlled trial in primary care to examine the mental health benefits of the *mobiletype *program. Patients aged 14 to 24 years were recruited from rural and metropolitan general practices. GPs identified and referred eligible participants (those with mild or more mental health concerns) who were randomly assigned to either the intervention group (where mood, stress, and daily activities were monitored) or the attention comparison group (where only daily activities were monitored). Both groups self-monitored for 2 to 4 weeks and reviewed the monitoring data with their GP. GPs, participants, and researchers were blind to group allocation at randomisation. Participants completed pre-, post-, and 6-week post-test measures of the Depression, Anxiety, Stress Scale and an Emotional Self Awareness (ESA) Scale.

**Results:**

Of the 163 participants assessed for eligibility, 118 were randomised and 114 participants were included in analyses (intervention group n = 68, comparison group n = 46). Mixed model analyses revealed a significant group by time interaction on ESA with a medium size of effect suggesting that the *mobiletype *program significantly increases ESA compared to an attention comparison. There was no significant group by time interaction for depression, anxiety, or stress, but a medium to large significant main effect for time for each of these mental health measures. Post-hoc analyses suggested that participation in the RCT lead to enhanced GP mental health care at pre-test and improved mental health outcomes.

**Conclusions:**

Monitoring mental health symptoms appears to increase ESA and implementing a mental health program in primary care and providing frequent reminders, clinical resources, and support to GPs substantially improved mental health outcomes for the sample as a whole.

**Trial Registration:**

ClinicalTrials.gov NCT00794222.

## Background

Mental health problems are common in young people with 75% of disorders beginning in adolescence and adolescent onset posing a considerable risk factor for long term psychological problems [1]. Adolescence is therefore likely to be an important phase for early intervention with primary care identified as the target setting in the World Health Organisation strategy for mental health [2]. General Practitioners (GPs) are often the providers of first step interventions for mental health (i.e. screening, monitoring, and psychoeducation), initially managing mental health concerns within their own clinical practice, then becoming conduits or gatekeepers to second step and further mental health care services (i.e. psychotherapy, medication, hospitalisation) when necessary [3,4]. Nevertheless, detection and management of mental health problems in primary care remains a challenge particularly with young people [5]; it is estimated that GPs detect at best 50% of mental health disorders [6,7]. Furthermore, 5.7% of adolescents are diagnosed with major depressive disorder [8] and up to 30% of young people experience mild depressive symptoms [9,10]. New methods are needed that focus on the early stages of mental health problems before clinically diagnosable mental health disorders are identified.

Poor recognition of symptoms by young people creates a significant barrier to communicating, detecting, and receiving help for mental health problems [11]. Research suggests that most people do not recognise the symptoms of depression and are suspicious about effective treatments [12]. Doctor related barriers to detection and management of mental health symptoms include insufficient time for assessments, a lack of confidence in managing and treating mental health symptoms, and a lack of systematic approaches to identify and provide evidence-based interventions for psychological disorders [13]. Detection rates of psychological problems are not necessarily associated with GP level of training in mental health or adolescent health [7], suggesting that further GP training in recognising mental health disorders may not be the most effective avenue for increasing detection rates.

There is some evidence that computerised screening, via portable computers such as Personal Digital Assistants or hand held touch pads (e.g. iPad) are both acceptable to patients and physicians, and can increase detection rates of health risk behaviours such as poor nutrition or exercise [14,15]. Short duration self-monitoring programs involving the completion of homework diaries have had some success at reducing depressive symptoms [16] and can be run on mobile phones [17,18]. Mobile phones provide a unique avenue for early intervention of mental health problems as they are an ubiquitous accessory, with 100% market penetration in Australia and Britain, and 67% worldwide [19]. Involving technology, such as computers, the internet or mobile phones in mental health programs can engage and foster young people's involvement [20-22]. Daily monitoring of mental health symptoms across time (i.e. between appointments) via mobile phones may assist young people in reducing their symptoms of mental health problems before reaching clinically diagnosable disorders. Further, daily monitoring data in addition to clinical assessment may allow for greater matching of services to patient needs and enhance pathways to care when second step care is indicated. From the patient's perspective, there is evidence that self-monitoring, on its own, is a therapeutic activity via increasing self-awareness [23], particularly of one's emotions, and leading to positive behaviour change [24-26], and therefore in the context of first step mental health care in primary care settings may lead to therapeutic outcomes.

As the integration of "e-health" reforms into primary care are considered a top priority [27], we have developed a novel mobile phone mental health assessment and management tool, the Mobile Tracking Young People's Experiences (*mobiletype*) program [17,18], designed for use in primary care and other clinical settings [28]. The *mobiletype *program monitors a young person's mood, stress, coping strategies and daily activities a number of times per day, and their eating, sleeping, exercise patterns, and alcohol and cannabis use once per day. This information is then uploaded to GPs, via a secure website and displayed in summary reports for review [17]. Our pilot study suggests that young people will monitor their mental health symptoms for the purpose of reviewing this data with their doctor and that both doctor and young person find this a beneficial way of communicating information about mental health and that the *mobiletype *program assisted the doctor to understand their patient better [28].

The overall aim of this study was to investigate, via a randomised controlled trial, a number of suggested benefits found in our pilot studies of the *mobiletype *program. This RCT was conducted as an effectiveness trial, in which we were interested the utility of the *mobiletype *program in the real world primary care setting. This paper reports on the primary outcomes of the RCT, namely, the mental health outcomes. We hypothesised that the mental health outcomes of participants who complete the *mobiletype *program and review the data with their GP will be lower at post-test and 6 weeks post-test compared with those in the attention comparison group.

## Methods

### Study design

The data presented here are the primary outcome data from the *mobiletype *randomised controlled trial conducted from 2009 to 2011. This was a multi-centre, multi-regional, stratified (according to region), single blind, attention-controlled study with balanced (1:1) individual randomisation into parallel-groups. This study was conducted in Victoria, Australia in a manner to allow for strict adherence to CONSORT reporting guidelines [29].

### Recruitment

#### General practitioners

All general practitioners in the Goulburn Valley Region and Albury/Wodonga Regions were invited to participate in the study via the Regional Division of General Practice (support units that service clinical practices within a region). Regional participants were overrepresented in this sample as mental health problems are an increasing concern in rural areas. GPs in Melbourne were recruited via the local Divisions of General Practice. Clinics that listed an interest in adolescent health on the Melbourne General Practice Network (http://www.mgpn.com.au) were particularly targeted. Participating GPs were trained in using the *mobiletype *website and provided a study manual which included the study procedure and a thorough range of clinical support, including referral details of adolescent friendly allied health professionals and services, youth-friendly internet, email and phone support, and youth-focused psychoeducation handouts and worksheets on a range of mental health problems (this information was also available on the *mobiletype *website). Continuing professional development quality assurance points were available to GPs for their participation in the study. Weekly reminder faxes were sent to all participating GPs and fortnightly phone calls to the GPs clinic (with the aim of speaking to the participating GP, but this was not always possible) were made to remind doctors of the study and to provide an update on recruitment to the study.

#### Young people

To best approximate the real world primary care setting, the following inclusion criteria were set: (1) aged 14 to 24 years, (2) speak proficient English and (3) have a mild or more severe emotional/mental health issue as assessed by their GP, or indicated by a K10 Symptom score greater than 16 [30]. Participants were excluded if they had a severe psychiatric or medical condition that prevented them from complying with either the requirements of informed consent or study protocol.

#### Intervention

Version 4 of the *mobiletype *program was used as the intervention in this study which was created using Java Platform, Micro Edition, in-house by the Murdoch Childrens Research Institute. This program was written for use with multiple models of mobile phones and firmware. For the purposes of this trial participants were lent a study mobile phone with either the *mobiletype *intervention or comparison program downloaded onto it. Data from the program was uploaded to a secure website constructed and hosted by MCRI as well as being encrypted and stored on the mobile phones.

Participants were prompted to complete a *mobiletype *entry by an auditory signal/beep emitted from the mobile phone at random intervals in the blocks outlined in Table 1. If no report was completed the phone emitted one reminder signal after 5 minutes. Entries were time-coded and saved. Participants were also able to complete the program any time and were able to complete an entry between 10 pm and 8 am although no trigger was sent at this time. The night time entry (00:00-08:00) consisted of the same questions as the afternoon questions as shown in Table 1. Each report took approximately 1-3 minutes to complete.

**Table 1:** Modules included in each block of the *mobiletype *intervention and comparison programs.

	Intervention				Attention Comparison			
	
	Morning	Noon	Afternoon	Evening	Morning	Noon	Afternoon	Evening
	08:00-10:59	11:00-15:29	15:30-19:59	20:00-00:00	08:00-10:59	11:00-15:29	15:30-19:59	20:00-00:00
*MODULE*								
Current Activity	✓	✓	✓	✓	✓	✓	✓	✓
Stress	✓	✓	✓	✓				
Mood	✓	✓	✓	✓				
Alcohol Use		✓						
Cannabis Use		✓						
Sleep	✓				✓			
Diet				✓				✓
Exercise				✓				✓

##### Intervention group

The intervention group monitored themselves using the complete *mobiletype *program which assessed 8 areas of functioning as developed and pilotted in previous *mobiletype *studies [17,18], consisting of current activities, location, companions, mood, recent stressful events, responses to stressful events, alcohol use, cannabis use, quality and quantity of sleep, and quantity and type of exercise, and diet (meals, snacks, "junk-food," and "soft-drinks" consumed). Participants who responded in a manner that indicated they were at risk of self-harm or suicide activated the program's high-risk alert, which would automatically send an SMS to our on call psychologist/phone counsellor. The psychologist would then call the young person and assess the risk of self-harm and alert the participant's local community assistance team if necessary. The time of day each module assessing the eight areas was delivered varied as displayed in Table 1.

##### Comparison group

The attention comparison protocol was designed to provide a data collection process similar to the intervention group by controlling for the amount of time spent engaged in the research methodology and the attention given to them by health care professionals and research staff [31]. The comparison group monitored themselves using an abbreviated version of the *mobiletype *program that assessed only current activities, location, companions, quality and quantity of sleep, and quantity and type of exercise, and diet. Importantly, the modules pertaining mental health as per Table 1 (i.e. mood, stress, alcohol and cannabis use) were removed.

##### Summary reports

Data collected by the *mobiletype *program (for both intervention and comparison groups) on the mobile phone was sent via SMS to a secure website constructed and hosted by MCRI, where it was automatically collated and available for viewing. Data was also encrypted and stored within phones, and all study phones were factory reset upon collection. Each area of assessment was displayed in graphs (i.e. daily mood graphs) or in tables (i.e. daily alcohol intake). An individualised summary report of the data was written following structured prescriptive guidelines by the first author (registered psychologist), or the second author under the supervision of the first author and consisted of mood, stress and coping, maintaining wellbeing and useful resources and recommendations for the intervention group. The comparison group also received individualized summary reports consisting of maintaining wellbeing (about their sleep, daily activities, diet, and exercise) and useful resources and recommendations.

#### Outcome Measures

The primary outcome measure was the Depression, Anxiety, Stress Scale (DASS) [32] 21 item response form, which is Australian and has Australian norms and clinically validated ranges. A high score indicates greater depression, anxiety, or stress. Kauer et al. [23] proposed that self-monitoring may affect mental health by increasing emotional self-awareness (ESA). As there is no direct measure of ESA, a scale was created by adapting the 20-item Self Reflection and Insight Scale [33], the 10-item Ruminative Response Scale [34] and the 12-item Meta-Evaluation Scale [35]. The total ESA scale had 33 items (the scale is available from the second author), scores ranged from 1 to 132, with higher scores indicating more ESA, and had high internal consistency (Cronbach's alpha = .83). Also included in the questionnaire package was general demographic information, the Short-Form 12 Heath Survey [36], an adapted version of the AUDIT [37], substance use (adapted from the Victorian Adolescent Health Cohort Study [38]), The Adolescent Coping Scale General Short Form [39,40]. Doctor-patient rapport, patient satisfaction, and pathways to care were assessed with the General Practice Assessment Questionnaire communication subscale [41], The Session Rating Scale [42], and The Party Project's Exit Interview [43] assessed pathways to care being if the participant was prescribed medications, referred to a health professional, referred for further testing, scans and/or X-rays, or provided other advice and psychoeducation regarding mental health during the most recent medical review. The pre-test, post-test, and 6 week post-test questionnaire packages included all of the above measures.

At pre-test, the GPs completed a questionnaire adapted from Haller et al [7] assessing the participants presenting concern, their current diagnostic information such as duration and medication, severity of any physical and mental health symptoms, and pathways to care implemented in current appointment (medication prescribed, referrals to other health professionals, and other interventions). GPs' confidence in dealing with the patient was measured with an adapted version of the SHO Appraisal Form [44]. At post-test the above measures were repeated and specific feedback regarding the usefulness, accuracy, helpfulness, and impact of mobiletype program on clinical practice was sought.

#### Sample size

Recruitment of 200 participants was anticipated from 10 general practices. This sample size was based upon Cohen's [45] statistical testing for multiple regression with two independent variables (to account for the mediating variable and the outcome) to detect a medium effect with 80% power and a probability of a type I error of .05. A medium effect size was selected as this was thought to be clinically significant. The anticipated sample size of 200 was not met due to delays in recruitment during school holidays and the H1N1 influenza pandemic. As a result, a deadline was set for stopping recruitment, and a total of 118 participants were recruited.

#### Randomisation

Participants were randomised to either i) the *mobiletype *monitoring intervention program group or (ii) the attention comparison program group; both groups also received usual medical care. Randomisation was conducted electronically, set up by an in-house computer programmer using random seed generation at the individual-level and stratified according to area (Melbourne, Goulburn Valley, and Albury/Wodonga). Study mobile phones were allocated ID numbers within areas (i.e. Melbourne01, Melbourne02) and either the intervention or comparison *mobiletype *program was loaded consecutively in a blinded fashion according to the programmer's concealed randomisation list. The randomisation list was constructed for 100 Melbourne, 50 Goulburn Valley and 50 Albury/Wodonga participants. Researchers, participants, and GPs were blind to randomisation pre-test. GPs and participants became aware of the group allocation at the post-test when the summary reports were reviewed. This study had Royal Children's Hospital Human Research Ethics Committee approval (HREC: 28113) and was registered in ClinicalTrials.gov (Reference: NCT00794222).

### Procedure

#### Recruitment

In addition to treatment as usual, GPs screened their patients for eligibility to the study, organised an appointment for interested participants with a research assistant using an online booking form, a faxed referral form, or by phone and completed a pre-test questionnaire. Participants met with a *mobiletype *research assistant within 5 days of referral to learn the study process, complete consent forms, the pre-test questionnaire package, review the *mobiletype *program and other features of the phone, and complete a practice entry of the *mobiletype *program. To protect doctor-patient confidentiality, parental consent was only sought when parents were present during the GP consultations; this process was approved by the Royal Children's Hospital Human Research Ethics Committee. Participants were provided with a study manual that described the research procedure and offered trouble-shooting tips.

#### Mobile phone monitoring period

All participants borrowed a Sony Ericsson Z750i mobile phone containing the *mobiletype *program for the study period. Information regarding the development and testing of the *mobiletype *program has been previously published [18,46]. Participants were requested to complete at least two *mobiletype *entries a day until they returned for their medical review in 2-4 weeks; participants and GPs were advised that 2-4 weeks was the ideal monitoring period. Participants were given a SIM card containing $30 in credit as partial reimbursement for their time and phone credit used.

##### Post-test review, 6-week post-test, and 6 month post-test assessments

Upon completion, participants reviewed the self-monitoring data with their GP on the *mobiletype *website. Young people completed a post-test assessment immediately following this appointment, again at six weeks and six months after this post-test review (6-month post-tests not included in the current analysis). GPs completed a post-test questionnaire immediately after the appointment. Questionnaires were completed online, over the phone with a research assistant, or via a mailed hardcopy survey. Participants were given a $20 gift card for each follow up survey completed (maximum of $60 for all questionnaires completed).

#### Analyses

Initially, to ascertain differences between groups in ESA, an intention to treat (ITT) mixed model analysis was conducted using SPSS v17.0.0 with the MIXED procedure. Survey time was entered as a continuous variable in weeks (0, 3, and 9). Subsequent to this, ITT mixed model analyses were again employed to test the primary mental health outcome. As planned contrasts were pivotal to interpretation of the mental health outcomes, the survey time was entered as categorical variable for these analyses. The mixed model method uses all available data, all participants at point of randomisation, without losing any cases, assuming that data is missing at random (MAR) [47]. All mixed models employed restricted maximum likelihood estimation method, and included subject identification number as a random effect and employed the time variable to create individual random slopes.

In terms of clustering, due to the number of GPs involved and the relatively small clusters within GPs, clustering at the GP did not provide a better approximation of the intraclass correlation coefficient than at the individual level. Geographic region was then considered as a proxy clustering variable, which returned a very low intraclass correlation coefficient of 0.007. As region clustering did not significantly contribute to the mixed model, clustering was not included. Multiple comparison adjustments (Bonferroni post-hoc style) were applied to subsequent a priori contrasts within the mental health mixed models, and Cohen's *d *measured the effect sizes of these contrasts [45].

The above analyses were repeated using a minimum effective dosage approach which only included participants who completed the minimum participation in the program (completing the recommended level of *mobiletype *entries: at least two entries per day for 14 days).

## Results

### Recruitment

**Figure 1:**
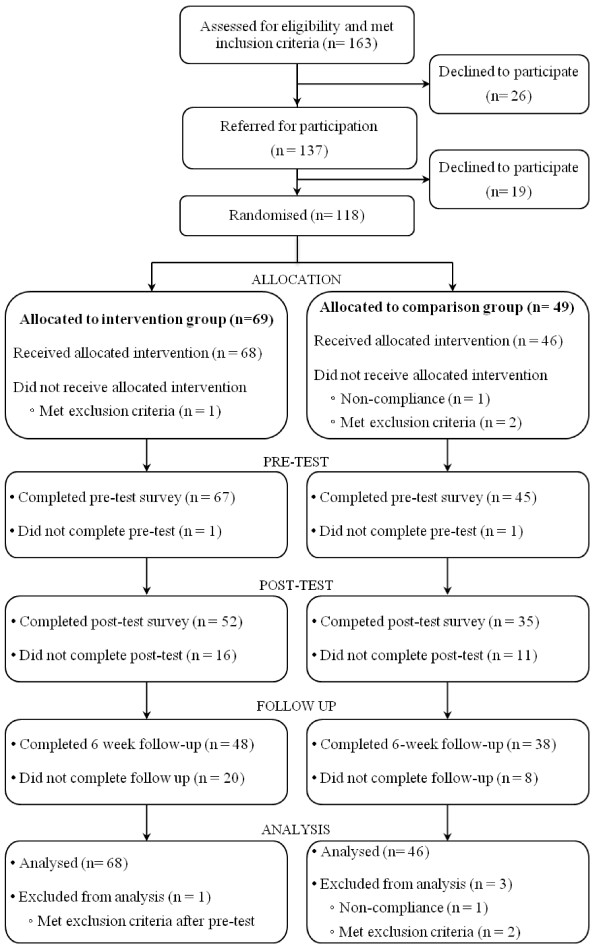
**Flow diagram of the study process**.

Data collection took place between the 16^th ^April 2009 and 28^th ^January 2011. Of the 103 GPs who agreed to participate, 35 actively recruited young people for the study. These contributing GPs were from 26 different practices in the three recruitment areas: 12 in greater Melbourne, 7 in Albury/Wodonga and 7 in the Goulburn Valley, resulting in an overrepresentation of general practices recruited in rural areas; 75% of Australian general practices are located in capital cities and suburbs [48]. Only 0.1% of Victorians live in remote areas and therefore were not targeted in this study [49]. As seen in Figure 1, 137 young people accepted the invitation to join the study, of whom 118 began the recruitment process. Four participants were excluded post randomisation (2 became too unwell to participate, one was incarcerated, and one gave invalid responses to all pre-test measures), resulting in a final sample of 114 young people which was sufficient to detect the primary aim of a medium-sized indirect effect [50]. Due to a failure to recruit the expected sample of 200 participants, there are different numbers of participants in the comparison and intervention groups, however, a test of the binomial distribution indicated that this difference was not significant with 69 out of 118 participants randomly allocated to the intervention group (*P *= .080). The total number of participants assessed for eligibility was difficult to establish as GPs rarely recorded information about patients who met the inclusion criteria and were either not approached to participate or who declined when invited to participate. Therefore the number of patients assessed for eligibility presented in Figure 1 is likely to be underrepresented.

In total, 63.2% of participants (72/114) completed all questionnaires and 84.2% of participants (96/114) completed questionnaires at two or more time points; several *t-*tests and χ^2 ^tests were conducted with no significant differences found between participants who completed all questionnaires and those who missed questionnaires. Therefore all 114 participants were included in the analyses. Three out of 114 participants (2.6%) did not complete the pre-test questionnaire, but went on to complete the *mobiletype *entries and post-tests. Sixteen participants (14%) were considered lost to follow-up, as they did not complete both the post-test and the 6 week post-test questionnaires.

### Demographics

No statistically significant differences in demographic information were found between the intervention and comparison group on any pre-test measures, as seen in Table 2.

**Table 2:** General demographics of participants in the comparison and intervention groups.

	Comparison Group *N *(%)	Intervention Group *N *(%)	*P *
**Total Number**	46 (40.4%)	68 (59.60%)	
**14 days completed**	28 (60.9%)	36 (52.9%)	.330
***Area***			
**Melbourne**	14 (30.4%)	28 (42.6%)	.265
**Goulburn Valley**	21 (45.7%)	21 (29.4%)	
**Albury/Wodonga**	11 (23.9%)	19 (27.9%)	
***Male Participants***	17 (37.0%)	15 (22.1%)	.082
***Mean (SD) Age, years***	17.4 (3.2)	18.5 (3.2)	.059
***Ethnic Identification***^**a**^	4 (9.1%)	10 (22.7%)	.365
***Employment***			
**Employed**	7 (15.2%)	18 (26.5%)	.212
**Unemployed**	4 (8.7%)	9 (13.2%)	
**Student**	35 (76.1%)	41 (60.3%)	
***Drug related items***^**a**^			
**Ever had alcohol**	38 (86.4%)	59 (88.1%)	.792
**Ever been drunk**	31 (70.5%)	53 (79.1%)	.299
**Ever had a cigarette**	25 (56.8%)	38 (56.7%)	.992
**Ever tried marijuana**	18 (40.9%)	33 (49.3%)	.388
**Ever tried other^b ^drugs**	10 (22.7%)	26 (38.8%)	.077

Note:^a ^n = 111,^b^sedatives, tranquilizers, amphetamines, analgesics, inhalants, cocaine, LSD and heroin.

Participants in the intervention group completed an average of 3.3 *mobiletype *entries each day (SD = 1.4, range 1-8 per day) and completed the program from one to 34 days with a mean of 14. 6 days completed (SD = 6.3). In the comparison group, participants completed an average of 4 *mobiletype *entries per day (SD = 1.8, range 1-12), and completed the program for eight to 25 days with a mean of 15.2 days completed (SD = 4.4). The minimum effective dose of the program was considered to be completing the *mobiletype *program two times a day for at least 14 days. As can be seen in Table 2, 36 (52.9%) participants in intervention and 28 (60.9%) in comparison received a minimum dose.

### Missingness

In order to satisfy the mixed model assumption of MAR, baseline demographics variables were compared between those with available and those with missing DASS outcomes at the post-test (30/114) and at the 6-week post-test (27/114) periods. A significant association was found between immediate follow-up survey completion and cigarette smoking, χ^2^(1) = 3.92, *p *= .048, with a higher proportion of those missing (72.41%) having smoked a cigarette than those completing the survey (51.2%). This was again found in the 6-week post-test, χ^2^(1) = 10.74, *p *= .001, with 84.62% of those missing the survey period reporting previously having smoked a cigarette, compared to 48.24% of those who completed the survey. No other significant associations/differences were found at either survey period.

### Outcome-Intention To Treat

Observed ESA and DASS mean differences were assessed at each time point using independent samples *t*-test, as shown in Table 3. A significant difference was found in observed ESA scores between groups at the 6-week post-test, with the intervention group mean ESA 6.6 points higher than the comparison, *t*(80) = 2.60, *p *= .011. Also, the intervention group reported significantly higher stress than the comparison group at pre-test, with a mean difference of 3.4, *t*(109) = 2.06, *p *= .042

**Table 3:** Observed means, standard deviations, 95% confidence intervals, sample size and mean differences f or each group at pre-, post- and 6-week post-test.

	Comparison Group			Intervention Group			Difference		
	*N*	*M (SD)*	95% CI	*N*	*M (SD)*	95% CI	*M diff*	*P*	*d*
***ESA***									
**Pre-test**	42	60.6 (11.9)	56.9-64.3	62	61.7 (12.1)	58.7-64.8	1.1	.65	0.09
**Post-test**	32	63.1 (11.1)	59.1-67.1	46	64.7 (10.9)	60.9-67.4	1.0	.69	0.09
**6-week**	35	62.2 (11.6)	58.2-66.1	47	68.9 (11.2)	65.5-72.1	6.6	.01	0.58
***Depression***									
**Pre-test**	44	19.4 (10.9)	16.1-22.7	67	20.4 (11.0)	17.8-23.1	1.0	.63	0.09
**Post-test**	33	15.2 (8.9)	12.1-18.3	50	16.3 (10.8)	13.3-19.4	1.1	.63	0.11
**6-week**	36	12.5 (11.8)	8.5-16.5	50	13.5 (10.5)	10.5-16.5	1.0	.69	0.09
***Anxiety***									
**Pre-test**	43	11.1 (8.1)	8.6-13.6	67	14.1 (9.7)	11.7-16.5	3.1	.09	0.26
**Post-test**	33	10.5 (8.0)	7.7-13.3	50	11.2 (9.1)	8.6-13.7	0.7	.73	0.25
**6-week**	35	10.4 (9.6)	7.1-13.7	50	9.8 (9.3)	7.1-12.4	0.6	.76	0.07
***Stress***									
**Pre-test**	44	16.9 (7.9)	14.5 -19.3	65	20.4 (8.9)	18.2-22.6	3.5	.04	0.40
**Post-test**	33	15.8 (8.0)	12.9-18.6	50	17.0 (9.4)	14.3-19.7	1.2	.53	0.14
**6-week**	35	13.1 (10.4)	9.6-16.7	50	15.2 (8.6)	12.8-17.6	2.1	.32	0.22

Note: Observed scores provided. *N *= number of participants used to calculate the mean (*M)*, standard deviation (*SD*) and 95% confidence interval *(CI)*, Mean difference calculated using *t*-tests, *d *= effect size (Cohen's *d *[51]).

Linear mixed models were applied to the ESA, the depression, anxiety, and stress subscales separately.

#### Emotional Self-Awareness

Results from the ESA mixed model analysis showed no significant group (β = 1.01, *P *= .635) or time main effects (β = 0.09, *P *= .675) but a significant interaction effect of group × time, (β = 0.59, *P *= .048), signifying different ESA patterns over time between groups. The observed means in Table 3 indicate that ESA increased in the intervention group from baseline to 6 week post-test, where as it remained the same from baseline to 6 week post-test in the attention comparison group. The size of effect of this difference at 6 weeks post-test was *d = *0.58, and according to Cohen [51], this is a medium size of effect.

#### Depression

**Figure 2:**
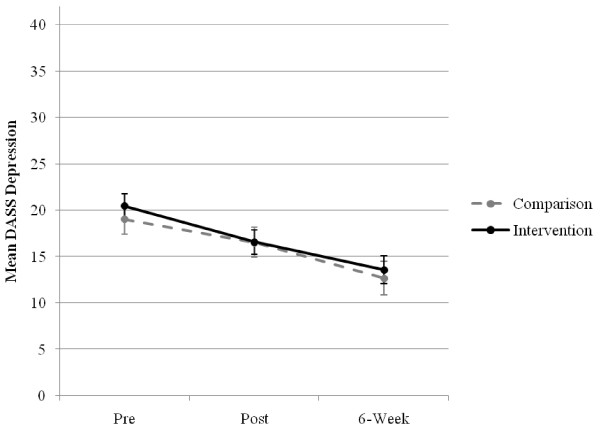
**Estimated marginal means (and standard errors) of depression scale for groups over time**.

There was no significant main effect for group, *F*(1, 110.48) = 0.19, *p *= .668), or interaction effect of group × time, *F*(2, 86.78) = 0.32, *p *= .729, but a significant main effect for time was found for depression, *F*(2, 86.78) = 14.63, *p *< .001. Subsequent contrasts of the estimated marginal means found a significant total group mean decrease of 6.59 points between baseline and 6-week post-test, *t*(96.32) = 5.26, *p *< .001, which was a medium size of effect (*d *= 0.53), a significant drop of 3.17 points from pre- to post-test, *t*(86.39) = 3.56, *p *= .001 (*d *= 0.37), and a further decrease of 3.42 from post- to 6-week post-test, *t*(81.21) = 2.97, *p *= .004 (*d *= 0.30). The estimated depression scale means and standard errors for each group across time are displayed in Figure 2.

#### Anxiety

**Figure 3:**
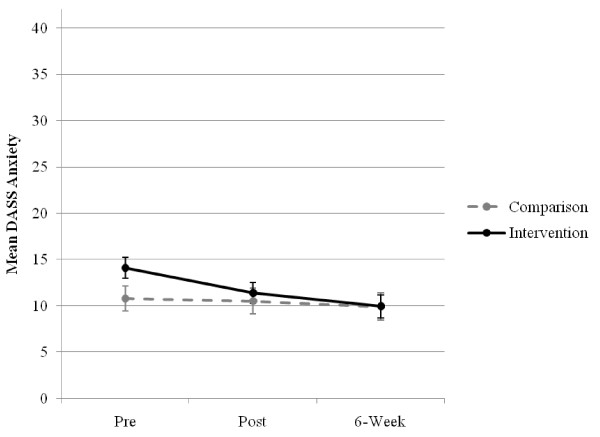
**Estimated marginal means (and standard errors) of the anxiety scale for groups over time**.

There was no significant main effect for group, *F*(1, 114.59) = 0.86, *p *= .356, or interaction effect of group × time, (*F*(2, 90.42) = 1.99, *p = .142*), but a significant main effect for time was found for the anxiety scale, *F*(2, 90.42) = 4.11, *p *= .020. Subsequent contrasts of the estimated marginal means found a significant total sample mean decrease 2.49 points from pre-test to 6-week post-test, *t*(93.35) = 2.76, *p *= .007 with a medium size of effect (*d *= 0.46), no significant further contrasts were significant. The estimated anxiety scale means and standard errors for each group across time are displayed in Figure 3.

#### Stress

**Figure 4:**
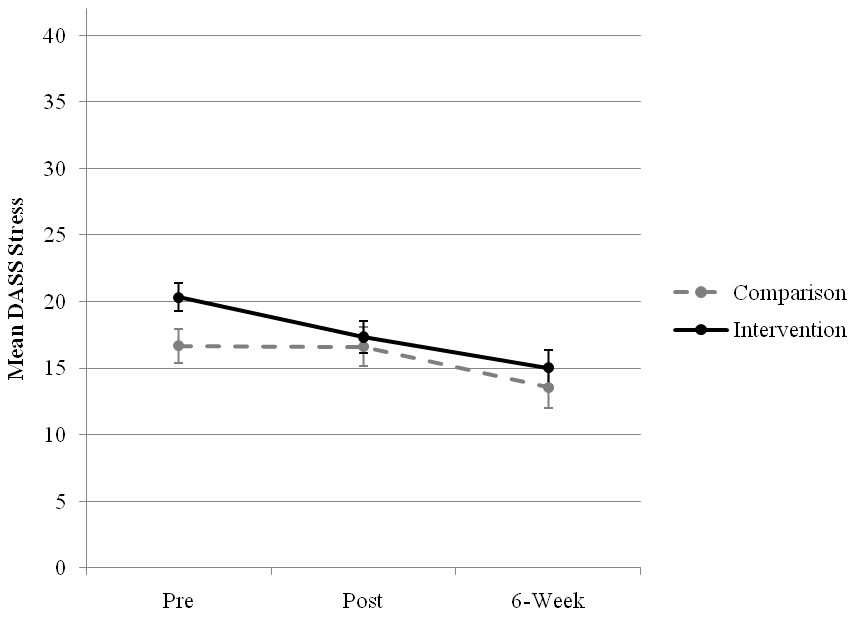
**Estimated marginal means (and standard errors) of the stress scale for groups over time**.

There was no significant main effect for group, *F*(1, 107.04) = 1.81, *p *= .181, or interaction effect of group × time, *F*(2, 86.41) = 1.52, *p = .225*. A significant effect for time was found, *F*(2, 86.41) = 8.38, *p *< .001, with post-hoc analyses showing a significant whole sample decrease of 4.19 points between pre- and 6-week post-test, *t*(91.63) = 4.08, *p *< .001 and an above medium size of effect (*d *= 0.57), but no significant differences between pre- and post-test, nor post- and 6-week post-test. The estimated stress scale means and standard errors for each group across time are displayed in Figure 4.

Secondary analyses were conducted with only those participants who completed 'a minimum dose' of monitoring (completion of 2 entries each day for at least 14 days). These results were similar to the ITT results with a significant effect of time for depression, *F*(2, 50.75) = 6.28, *p *= .004, with the size of effect for time being a 6.08 point decrease from pre- to 6-week post-test, *t*(58.02) = 3.53, *p *= .001 (*d *= 0.47), and no other significant effects for depression. The main effect of time for anxiety was no longer significant, and there were no other significant effects. There was a significant effect of time for stress, *F*(2, 50.05) = 4.21, *p *= .020, with a decrease of 4.25 points from pre- to 6-week post-test, *t*(54.29) = 2.89, *p *= .005 (*d *= 0.39), and no other significant effects for stress.

### Post-hoc Analyses

Post-hoc analyses were conducted to investigate the above findings that across the whole sample depression, anxiety and stress scale scores decreased by a medium size of effect from pre-test to 6 weeks post-test. One possible explanation is that the attention comparison was also an active intervention because participation in the trial lead to changes in GP management of mental health concerns for all participants. The Party Project's Exit Interview [43] at pre-test and post-test assessed pathways to care being if the participant was prescribed medications, referred to a health professional, referred for further testing, scans and/or X-rays, or provided other advice and psychoeducation regarding mental health during the medical review by their GP. For the sample as a whole, 91.7% (100/109) received at least one and 55.0% (60/109) received at least two "pathways to care" in the pre-test medical review. Interestingly, there was not one predominant pathway or intervention, 56.0% of the sample was prescribed medications, 54.1% referred to a health care professional, 38.5% received other advice or psychoeducation about mental health, and 25.7% were referred for further tests. This finding suggests that GPs were intervening regarding mental health for all participants at the pre-test medical review, rather than waiting for further information from the *mobiletype *program.

## Discussion

The aim of this study was to conduct a randomised controlled effectiveness trial to examine the mental health benefits of adding the mobiletype program and GP review of the mobiletype data to usual medical care of at-risk young people. The intervention group had an increase of ESA over time with a significant effect of the *mobiletype *program on ESA between pre- and 6-week post-test when compared to the attention comparison group. Results suggest that the self-monitoring intervention program increases young people's ESA during self-monitoring, between pre- and post-test, but this effect was not significant until 6 weeks after completion of the program. Nevertheless, the *mobiletype *intervention group's mental health outcomes did not improve significantly more than the attention comparison group at post-test or 6 weeks. For the sample as a whole, however, there was a substantial decrease in each of the depression, anxiety, and stress scores from pre-test to 6 weeks post-test and this decrease was a medium size of effect for each scale, suggesting that both groups improved in mental health outcomes substantially from pre-test to 6 weeks post-test. The decrease in mental health symptoms 6 weeks after the program may be explained by the effect of young people having increased their awareness of their emotions. A secondary outcomes paper from this study further explores the possible mediating effect of ESA on depressive symptoms [52].

Further investigation of the mental health outcomes was warranted because the size of the decrease in depression, anxiety, and stress symptoms was greater than one would expect from simple retest effects alone [53,54]. Post-hoc analyses of GP behaviour at pre-test suggested that in fact GPs were unexpectedly acting to manage mental health during the pre-test review in at least one manner for 92% of the participants, without waiting for further information from the *mobiletype *monitoring data, summary report and recommendations. As this was an effectiveness trial, GPs were instructed to simply add *mobiletype *to usual medical care: there was no specific instruction to wait for *mobiletype *data before referring, prescribing, or implementing other patient management strategies (nor would this have been ethical). In this study, GPs received frequent contact and reminders from the research team thus raising the saliency of youth mental in clinical care, and were also provided with comprehensive locally relevant clinical resources (referral sources and psychoeducation handouts). Further, as the general rate of return to follow up review appointments in primary care can be unpredictable particularly with young people [55,56], it is understandable that GPs felt the need to manage mental health symptoms when they first present rather than wait for further data and risk the possibility of the patient not returning. In this study we included an "attention comparison" group rather than wait-list control due to ethical considerations, thus increasing the testing rigour of the intervention, but also increasing the likelihood of non-specific placebo effects on outcome measures due to the attention comparison participants also receiving an intervention of sorts [57]. The results of this trial suggest that a self-monitoring program which monitors young people's mood, stress, and coping can increase young people's awareness of their emotions more than a program which only monitors general health factors. Self-monitoring may assist young people to become aware of emotions and stressors and therefore prepare themselves for more adaptive coping strategies. This study also suggests that engaging GPs in a mental health trial, providing frequent reminders, clinical resources and referral pathways, a patient self-monitoring program (as the attention comparison group also self-monitored) and assisting patients to return for medical reviews, leads to positive mental health outcomes for patients.

Furthermore, this RCT was conducted with a view of representing a wide variety of young people who visit GPs with a range of medical and psychological problems and severity of problems. Therefore the results of this study are applicable to this age group in general. Nevertheless, compared to data from the Australian Bureau of Statistics, the rural sample in this study is overrepresented, with 53.8% of general practices located in rural Victoria participating this this study compared to 24% of general practices located in rural Victoria in the population [48]. In addition, there was an overrepresentation of female patients with 80.4% female patients recruited in the current sample compared to 53% of females that seek treatment in general health care practices.

### Limitations

A cluster randomised controlled trial with a usual medical care control group in which GPs or clinics were randomised rather than individuals may have been a more appropriate design for this intervention. This design was rejected for the current study, however, in preference for a more rigorous attention comparison group and individual randomisation, as it would have been logistically impossible for GPs and participants to be blinded to randomisation at recruitment in a cluster RCT and there is considerable potential for the introduction of bias in unblinded cluster RCTs when inclusion to the study is based upon GP referral (i.e. comparison GPs may only refer less severe patients) and participant consent is required (i.e. comparison participants may be less likely to consent) [58]. The inclusion of a wait-list control or "usual medical care only" group in this study would have allowed for comparison and testing of the substantial decrease in depression, anxiety, and stress in the mobiletype and attention comparison groups to a more natural control group. Nevertheless, the pre-test pathways to care implemented at baseline by GPs may also have occurred for a treatment as usual group and may have decreased mental health symptoms in both groups, thereby reducing the power needed to detect a significant difference between the groups. A larger sample size, or a wait-list control group, would be needed to determine if there was a difference in depressive symptoms between groups [59,60]. Participant heterogeneity no doubt decreased the power of this trial, yet, a significant interaction effect was found for ESA. It appears, however, that the mental health intervention implemented by the GPs at baseline "trumped" or superseded any therapeutic effect of increasing ESA may have on mental health. As this was an effectiveness trial, the participant inclusion criteria were designed to be a best approximate to the real world primary care setting and hence there was a range of severity in mental health, familiarity of patients to GPs, and current mental health treatment programs. Finally, the random outcome of uneven groups due to cessation of recruitment before completion of the randomisation list also reduced the power of the study to find an effect between the intervention and attention comparison groups. A greater sample size and more even distribution between groups may have lead to more equivalent baseline means.

There has been much discussion internationally about the need to support GPs in the detection and management of mental health [61], with the WHO producing a number of reports and strategies papers regarding this [2]. Changing GP mental health related practice and patient outcomes in primary care has proven difficult with one review citing that only 21 out of 36 RCTs which implemented a number of either intensive plans, protocols, nurse led-care, and specialist assistance for mental health care in primary care lead to positive mental health outcomes for patients [61,62]. The current mobiletype study included many aspects of two large successful primary care RCT mental health interventions [63,64], for example, screening, clinician education, patient-specific reminders for appointments and patient care by the research team, and showed an overall mental health benefit for the sample as a whole and demonstrated that technology, particularly mobile phones, can be used in clinical settings and may provide GPs and young people with an avenue for combating the early symptoms of mental health problems before a clinically diagnosable disorder presents. Whilst this study does not demonstrate any additional benefits of monitoring specific mood, stress, coping, alcohol and cannabis use and general health factors compared to monitoring general health factors alone, further research using this methodology with larger sample sizes and a waitlist control seems warranted.

## Conclusions

We conducted the first RCT of a mobile phone application in the mental health assessment and management of youth mental health in primary care. We found that actively monitoring one's mental health symptoms using mobile phones led to increased emotional self-awareness. Further, that implementing a mental health program using technology in primary care and providing frequent reminders, clinical resources and support to GPs substantially improved mental health outcomes for the sample as a whole.

## Competing interests

The authors declare that they have no competing interests.

## Authors' contributions

SCR conceived of, designed, and implemented the study, she also participated in the analyses and drafted the manuscript. SK participated in the conception, design, and implementation of the study, participated in the analyses and contributed to the drafting of the manuscript. SJCH conducted the analyses and participated in drafting the manuscript. AHDC coordinated the implementation of the study and contributed to drafting the manuscript. ASK contributed to the design of the study, collected data for the study and contributed to drafting the manuscript. LAS and GCP both contributed to the design of the study and drafting of the manuscript. All authors read and approved the final manuscript.

## Pre-publication history

The pre-publication history for this paper can be accessed here:

http://www.biomedcentral.com/1471-2296/12/131/prepub

## References

[B1] Kessler RC Berglund P Demler O Jin R Merikangas KR Walters EE Lifetime prevalence and age-of-onset distributions of DSM-IV disorders in the national comorbidity survey replication Archives of General Psychiatry 2005 62 593 602 10.1001/archpsyc.62.6.593 15939837

[B2] WHO Integrating mental health into primary care: a global perspective 2008 Geneva: World Health Organization and World Organization of Family Doctors (Wonca)

[B3] van Straten A Seekles W van't Veer-Tazelaar NJ Beekman ATF Cuijpers P Stepped care for depression in primary care: what should be offered and how? Medical Journal of Australia 2010 192 11 Suppl S36 S39 20528706 10.5694/j.1326-5377.2010.tb03691.x

[B4] Hickie IB Groom GL McGorry PD Davenport TA Luscombe GM Australian mental health reform: time for real outcomes Medical Journal of Australia 2005 182 401 406 15850437 10.5694/j.1326-5377.2005.tb06759.x

[B5] Raine R Lewis L Sensky T Hutchings A Hirsch S Black N Patient determinants of mental health interventions in primary care British Journal of General Practice 2000 50 457 620 625 11042912 PMC1313771

[B6] Hickie IB Davenport TA Naismith SL Scott EM Conclusions about the assessment and management of common mental disorders in Australian general practice Medical Journal of Australia 2001 175 S 52 55 11556438 10.5694/j.1326-5377.2001.tb143791.x

[B7] Haller DM Sanci LA Sawyer SM Patton GC The identification of young people's emotional distress: A study in primary care British Journal of General Practice 2009 59 560 e61 e70 10.3399/bjgp09X419510 19275825 PMC2648934

[B8] Costello EJ Erkanli A Angold A Is there an epidemic of child or adolescent depression? Journal of Child Psychology and Psychiatry 2006 47 12 1263 1271 17176381 10.1111/j.1469-7610.2006.01682.x

[B9] Andrews G Szabo M Burns J Preventing major depression in young people British Journal of Psychiatry 2002 181 460 462 10.1192/bjp.181.6.460 12456512

[B10] Lewinsohn PM Hops H Roberts RE Seeley JR Andrews JA Adolescent psychopathology: I. Prevalence and incidence of depression and other DSM-III--R disorders in high school students Journal of Abnormal Psychology 1993 102 1 133 144 8436689 10.1037//0021-843x.102.1.133

[B11] Rickwood D Cavanagh S Curtis L Sakrouge R Educating young people about mental health and mental illness: evaluating a school-based programme International Journal of Mental Health Promotion 2004 6 4 13

[B12] Jorm AF Mental health literacy. Public knowledge and beliefs about mental disorders British Journal of Psychiatry 2000 177 396 401 10.1192/bjp.177.5.396 11059991

[B13] Hickie IB Fogarty AS Davenport TA Luscombe GM Burns J Responding to experiences of young people with common mental health problems attending Australian general practice Medical Journal of Australia 2007 187 7 Suppl S47 S52 17908026 10.5694/j.1326-5377.2007.tb01337.x

[B14] Stevens J Kelleher KJ Gardner W Chisolm D McGeehan J Pajer K Buchanan L Trial of computerized screening for adolescent behavioral concerns Pediatrics 2008 121 6 1099 1105 10.1542/peds.2007-1878 18519478

[B15] Olson AL Gaffney CA Hedberg VA Gladstone GR Use of inexpensive technology to enhance adolescent health screening and counseling Archives of Pediatrics & Adolescent Medicine 2009 163 2 172 177 10.1001/archpediatrics.2008.533 19188650

[B16] Stice E Shaw H Bohon C Marti CN Rohde P A meta-analytic review of depression prevention programs for children and adolescents: factors that predict magnitude of intervention effects Journal of Consulting and Clinical Psychology 2009 77 3 486 503 19485590 10.1037/a0015168 PMC2758769

[B17] Kauer S Reid S Sanci L Patton G Investigating the utility of mobile phones for collecting data about adolescent alcohol use and related mood, stress and coping behaviours: Lessons and recommendations Drug & Alcohol Review 2009 28 1 25 30 10.1111/j.1465-3362.2008.00002.x 19320672

[B18] Reid SC Kauer SD Dudgeon P Sanci LA Shrier LA Patton GC A mobile phone program to track young people's experiences of mood, stress and coping. Development and testing of the mobiletype program Social Psychiatry and Psychiatric Epidemiology 2009 44 6 501 507 10.1007/s00127-008-0455-5 19011723

[B19] World Telecommunications/ICT Indicators Electronic Database http://www.itu.int

[B20] Walsh SP White KM Young RM Over-connected? A qualitative exploration of the relationship between Australian youth and their mobile phones Journal of Adolescence 2008 31 1 77 92 10.1016/j.adolescence.2007.04.004 17560644

[B21] Zhao S Grasmuck S Martin J Identity construction on Facebook: digital empowerment in anchored relationships Computers in Human Behavior 2008 24 1816 1836 10.1016/j.chb.2008.02.012

[B22] Hollon SD Muñoz RF Barlow DH Beardslee WR Bell CC Bernal G Clarke GN Franciosi P Kazdin AE Kohn L Psychosocial intervention development for the prevention and treatment of depression: promoting innovation and increasing access Biological Psychiatry 2002 52 6 610 630 10.1016/S0006-3223(02)01384-7 12361671

[B23] Kauer S Reid S Jackson H Jorm T The phenomenon of insight and preliminary findings for preventing depression: self-monitoring via mobile phones 'ASPR Poster Presentations' Australian and New Zealand Journal of Psychiatry 2007 41 A496

[B24] Kazantzis N Power to detect homework effects in psychotherapy outcome research Journal of Consulting and Clinical Psychology 2000 68 1 166 170 10710851

[B25] Abueg FR Colletti G Kopel SA A study of reactivity: the effects of increased relevance and saliency of self-monitored smoking through enhanced carbon monoxide feedback Cognitive Therapy and Research 1985 9 321 333 10.1007/BF01183851

[B26] Ewart CK Self-observation in natural enviroments: reactive effects of behavior desirability and goal-setting Cognitive Therapy and Research 1978 2 39 56 10.1007/BF01172511

[B27] Van Der Weyden MB General practice and e-health reform Medical Journal of Australia 2010 193 2 69 20642403 10.5694/j.1326-5377.2010.tb03799.x

[B28] Reid S Kauer S Sanci L Patton G Please turn your mobiles on in our clinic: a mobile phone mental health monitoring program and website interface for detection and management of adolescent mental health in clinical settings 'ASPR Poster Presentations' Australian and New Zealand Journal of Psychiatry 2007 41 A499

[B29] Altman DG Schilz KF Moher D Egger M Davidoff F Elbourne D Gotzsche PC Lang T The revised CONSORT statement for reporting randomised trials: explanation and elaboration Annals of Internal Medicine 2001 134 8 663 694 11304107 10.7326/0003-4819-134-8-200104170-00012

[B30] Andrews G Slade T Interpreting scores on the Kessler Psychological Distress Scale (K10) Australian and New Zealand Journal of Public Health 2001 25 6 494 497 10.1111/j.1467-842X.2001.tb00310.x 11824981

[B31] LIndquist R Wyman JF Talley KMC Findorff MJ Gross CR Design on control-group conditions in clinical trials of behavioral interventions Journal of Nursing Scholarship 2007 39 3 214 221 10.1111/j.1547-5069.2007.00171.x 17760793

[B32] Lovibond PF Lovibond SH The structure of negative emotional states: comparison of the Depression Anxiety Stress Scales (DASS) with the Beck Depression and Anxiety Inventories Behaviour Research and Therapy 1995 33 3 335 343 10.1016/0005-7967(94)00075-U 7726811

[B33] Grant AM Franklin J Langford P The Self-Reflection and Insight Scale: a new measure of private self-consciousness Social Behavior and Personality 2002 30 8 821 836 10.2224/sbp.2002.30.8.821

[B34] Treynor W Gonzalez R Nolen-Hoeksema S Rumination reconsidered: a psychometric analysis Cognitive Therapy and Research 2003 27 247 259 10.1023/A:1023910315561

[B35] Mayer JD Stevens AA An emerging understanding of the reflective (meta-) experience of mood Journal of Research in Personality 1994 28 351 373 10.1006/jrpe.1994.1025

[B36] Ware JE Kosinski M Keller SD A 12-Item Short-Form Health Survey: construction of scales and preliminary tests of reliability and validity Medical Care 1996 34 3 220 233 10.1097/00005650-199603000-00003 8628042

[B37] Saunders JB Aasland OG Babor TF de la Fuente JR Grant M Development of the Alcohol Use Disorders Identification Test (AUDIT): WHO Collaborative Project on Early Detection of Persons with Harmful Alcohol Consumption--II Addiction 1993 88 6 791 804 10.1111/j.1360-0443.1993.tb02093.x 8329970

[B38] Patton GC Hibbert M Rosier MJ Carlin JB Caust J Bowes G Patterns of common drug use in teenagers Australian Journal of Public Health 1995 19 4 393 399 7578541 10.1111/j.1753-6405.1995.tb00392.x

[B39] Frydenberg E Lewis R Adolescent coping: the different ways in which boys and girls cope Journal of Adolescence 1991 14 2 119 133 10.1016/0140-1971(91)90025-M 1918514

[B40] Frydenberg E Lewis R Boys play sport and girls turn to others: age, gender and ethnicity as determinants of coping Journal of Adolescence 1993 16 3 253 266 10.1006/jado.1993.1024 8282897

[B41] Mead N Bower P Roland M The General Practice Assessment Questionnaire (GPAQ)-development and psychometric characteristics BMC Family Practice 2008 9 13 10.1186/1471-2296-9-13 18289385 PMC2277420

[B42] TalkingCure.com

[B43] PARTY Project http://www.party.unimelb.edu.au

[B44] Appraisal Record of Senior House Officer http://www.webcitation.org/query.php?url=http://www.rcplondon.ac.uk/pubs/contents/304a5fdc-cc4e-4d3e-8270-49403e35fe8e.pdf&refdoi=10.1186/1472-6920-8-22

[B45] Cohen J A power primer Psychological Bulletin 1992 112 1 155 159 19565683 10.1037//0033-2909.112.1.155

[B46] Reid S Patton G Sanci L Kauer S Understanding how young people cope with distress: the development of a mobile phone momentary sampling program (Mobile_TYPE) Acta Neuropsychiatrica 2006 18 268 10.1017/S0924270800030738 27397230

[B47] Hedeker D Gibbons RD Application of random-effects pattern-mixture models for missing data in longitudinal studies Psychological Methods 1997 2 1 64 78

[B48] 8570.0 Health Care Services, 2009-10: Regional characteristics http://www.abs.gov.au/AUSSTATS/abs@.nsf/DetailsPage/8570.02009-10?OpenDocument

[B49] 1301.0 Year Book Australian, 2004: How many people live in Australia's remote areas? http://www.abs.gov.au/ausstats/abs@.nsf/Previousproducts/1301.0Feature Article22004?opendocument&tabname=Summary&prodno=1301.0&issue=2004&num=&view=

[B50] Fritz MS MacKinnon DP Required sample size to detect the mediated effect Psychological Science 2007 18 3 233 239 10.1111/j.1467-9280.2007.01882.x 17444920 PMC2843527

[B51] Cohen J Statistical power analysis for the behavioral sciences 1988 Hillside, NJ: Erlbaum

[B52] Kauer SD Reid SC Crooke AHD Khor A Hearps SJC Jorm AF Sanci L Patton G Self-monitoring using Mobile Phones in the Early Stages of Adolescent Depression: A Randomised Controlled Trial with an Attention Comparison Group to Examine The Mediating Effect of Emotional Self-Awareness Journal of Medical Internet Research in press 10.2196/jmir.1858 PMC3414872 22732135

[B53] Longwell BT Turax P The differential effects of weekly, monthly, and bimonthly administrations of the Beck Depression Inventory-II: psychometric properties and clinical implications Behavior Therapy 2005 36 265 275 10.1016/S0005-7894(05)80075-9

[B54] Jorm AF Duncan-Jones P Scott R An analysis of the re-test artefact in longitudinal studies of psychiatric symptoms and personality Psychol Med 1989 19 2 487 493 10.1017/S0033291700012514 2788291

[B55] Walker Z Townsend J Oakley L Donovan C Smith H Hurst Z Bell J Marshall S Health promotion for adolescents in primary care: randomised controlled trial BMJ 2002 325 7363 524 10.1136/bmj.325.7363.524 12217993 PMC121334

[B56] Churchill R Allen J Denman S Williams D Fielding K von Fragstein M Do the attitudes and beliefs of young teenagers towards general practice influence actual consultation behaviour? Br J Gen Pract 2000 50 461 953 957 11224965 PMC1313880

[B57] Finniss DG Kaptchuk TJ Miller F Benedetti F Biological, clinical, and ethical advances of placebo effects Lancet 2010 375 9715 686 695 10.1016/S0140-6736(09)61706-2 20171404 PMC2832199

[B58] Puffer S Torgerson DJ Watson J Evidence for risk of bias in cluster randomised trials: review of recent trials published in three general medical journals British Medical Journal 2003 327 785 789 10.1136/bmj.327.7418.785 14525877 PMC214092

[B59] Fritz MS MacKinnon DP Required sample sizr to detect the mediated effect Psychological Science 2007 18 3 233 239 10.1111/j.1467-9280.2007.01882.x 17444920 PMC2843527

[B60] Hayes AF Beyond Baron and Kenny: Statistical mediation analysis in the new milennium Communication Monographs 2009 76 4 408 420 10.1080/03637750903310360

[B61] Gilbody S Whitty P Grimshaw J Thomas R Educational and organizational interventions to improve the management of depression in primary care: a systematic review JAMA 2003 289 23 3145 3151 10.1001/jama.289.23.3145 12813120

[B62] WHO The World Health Report 2001: Mental Health: New Understanding, New Hope 2001 Geneva: World Health Organisation

[B63] Wells KB Sherbourne C Schoenbaum M Duan N Meredith L Unützer J Miranda J Carney MF Rubenstein LV Impact of disseminating quality improvement programs for depression in managed primary care: a randomized controlled trial JAMA 2000 283 2 212 220 10.1001/jama.283.2.212 10634337

[B64] Rubenstein LV Jackson-Triche M Unützer J Miranda J Minnium K Pearson ML Wells KB Evidence-based care for depression in managed primary care practices Health Aff (Millwood) 1999 18 5 89 105 10.1377/hlthaff.18.5.89 10495595

